# Supervisors’ Visual Attention Allocation Modeling Using Hybrid Entropy

**DOI:** 10.3390/e21040393

**Published:** 2019-04-12

**Authors:** Haifeng Bao, Weining Fang, Beiyuan Guo, Peng Wang

**Affiliations:** State Key Lab Rail Traff Control & Safety, School of Mechanical, Electronic and Control Engineering, Beijing Jiaotong University, 100044 Beijing, China

**Keywords:** attention allocation, attention behavior, hybrid entropy, information entropy

## Abstract

With the improvement in automation technology, humans have now become supervisors of the complicated control systems that monitor the informative human–machine interface. Analyzing the visual attention allocation behaviors of supervisors is essential for the design and evaluation of the interface. Supervisors tend to pay attention to visual sections with information with more fuzziness, which makes themselves have a higher mental entropy. Supervisors tend to focus on the important information in the interface. In this paper, the fuzziness tendency is described by the probability of correct evaluation of the visual sections using hybrid entropy. The importance tendency is defined by the proposed value priority function. The function is based on the definition of the amount of information using the membership degrees of the importance. By combining these two cognitive tendencies, the informative top-down visual attention allocation mechanism was revealed, and the supervisors’ visual attention allocation model was built. The Building Automatic System (BAS) was used to monitor the environmental equipment in a subway, which is a typical informative human–machine interface. An experiment using the BAS simulator was conducted to verify the model. The results showed that the supervisor’s attention behavior was in good agreement with the proposed model. The effectiveness and comparison with the current models were also discussed. The proposed attention allocation model is effective and reasonable, which is promising for use in behavior analysis, cognitive optimization, and industrial design.

## 1. Introduction

With the improvement in automation technology, the role of humans in complicated control systems is changing from that of operators to supervisors [1]. More and more information is being displayed on human–machine interfaces, but human attention ability is limited. Therefore, the limited attention resources of supervisors are precious and important. Most system failures and operational accidents are due to the lack of visual attention to relevant information [2]. Analyzing the visual attention behaviors and revealing the visual attention allocation mechanism are important for the design and evaluation of human–machine interfaces (HMIs). HMIs with an ergonomic design that align with the attention behaviors of the supervisors are useful for system safety, error evaluation, and accident prevention [3,4,5].

Attention behavior has many aspects, such as hearing, vision, and touch. Among them, vision is important to supervisors during the task of monitoring. Humans have a complex selective visual attention behavior that scans the scene both in a rapid, bottom-up, salience-driven manner as well as a slower, top-down, task-dependent manner [6]. The visual attention to bottom-up salient information is a rapid process that has limited effects on task-dependent attention allocation. Supervisory behavior is a long-term attention allocation mechanism for familiar scenes. The top-down task-driven factors occupy the majority of the attention strategy during supervisory tasks. 

Many factors can affect attention behaviors. Salience-driven factors depend on visual features such as salience, blinking, shape, and colors [7,8,9]. The task-driven factors in the supervisory task depend on the task features such as urgency, expectation, effort, and value [10,11,12,13]. Supervisors always comprehensively consider the above task-factors during the task process, then establish the priority of the information. In the task, the importance of the displayed information is mainly considered by the supervisors. Matsuka proved that human learners do not always optimize attention; one reason they fail to do so is that, under certain conditions, the cost of information retrieval or use may affect the attention strategy adopted by the learners [14]. Therefore, in familiar procedural tasks, supervisors acquire system information based on their experience and previously acquired knowledge due to the top-down attention strategy.

The determination of information priorities is complicated and fuzzy in the cognitive process. The uncertainty of the information may produce significant anxiety in supervisors, who tend to pay attention to the information sections that can reduce that indeterminacy. The attention to information is a reduction of the entropy of the HMI. This complicated cognitive behavior was described as mental entropy processing by Wanyan [15]. Even though mental entropy theory has some limitations, it was used successfully in modeling the cognitive process for information processing in the human brain. Supervisors tend to pay attention to the visual section which has a higher information value. Therefore, the membership degrees of the importance of the information sections based on fuzzy theory could be feasibly used to quantify its value. These two selective cognitive mechanisms have been shown to synergistically affect attention behaviors [16,17]. 

Efficient HMIs help their users accomplish their tasks with minimal workload and fatal errors. The visual attention model is useful for the design and optimization of these interfaces [18,19]. The layout of a T-type HMI on aircraft was constructed by Fitts by analyzing the pilots’ visual attention behavior [20]. The visual attention model predicted the users’ selective attention behavior in supervisory tasks, which was beneficial in staff training [21]. One important aspect of on-the-job training of supervisors is to make them pay attention to the right section at the right time. Using the model, the researchers evaluated the mental workload and situation awareness of the user, which provided information about the conditions of the user’s current mental status [22,23]. This model can also guide task analysis and contribute to task optimization [12]. Overall, the visual attention allocation model is useful and promising.

At present, evaluating visual attention is easily accomplished by tracking eye gaze in or after the supervisory task [24]; however, predicting the visual attention allocation behaviors before the task is challenging. We aimed to build an effective, accurate, and quantified model in visual attention allocation based on the related works.

## 2. Related Works

In previous studies, researchers proposed many valuable attention allocation models to predict the supervisory behavior of supervisors in informative HMIs. Based on saliency-based image recognition, the predictive attention model was built which considered the bottom-up attention mechanism of humans [6,9,25,26]. The observable information on the screen could be recognized using deep learning to predict the attention behavior [27,28,29]. These bottom-up models help us reveal the basic attention mechanism that how humans react to images. Wickens developed the SEEV model of scanning behavior considering the task-driven factors [10,11,12]. This model considers the salience, effort, expectancy, and value (SEEV) associated with each visual section. The model was improved to NT-SEEV, to predict the notice ability (NT for notice) of events that occurred in the context of routine task-driven scanning across large-scale visual environments [30]. Many researchers worked on the quantitation and computation of multiple factors in SEEV [31,32,33]. SEEV and its improved models consider both the bottom-up and top-down attention mechanisms of humans. However, due to the different chosen factors and computational methods, the results of the above models have varied significantly. 

Some researchers computed attention allocation using gaze data based on fuzzy theory [34,35]. However, this involved a post analysis method that could not predict the attention allocation strategy. Senders considered the human operator as a monitor and controller in the system [36]. The model argues that humans are information processors and supervisory behavior is a data processing process. The model describes the strategy of humans when selecting their attention focus in an informative HMI. Sheridan distinguished the time interval of the supervisor when processing the information and the proposed model assumed that the operator controls the most valuable information with each sample [37]. Visual information processing is fuzzy in the human brain. Lin introduced a novel fractional-order chaotic phase synchronization model for visual selection and shifting [38]. The model uses two chaotic network layers to simulate the human cognitive system and solves the processing of the natural image in the brain, which was useful for the proposed model in this article. Junshan used multiscale entropy analysis of human operating behavior, which is a post-analysis method to determine the human dynamics [39]. Pan extended the influence model to incorporate dynamical parameters to a social system, which allowed us to uncover important shifts between actors. The model is instructive in attention shift behavior [40].

Based on the above work, Matsui researched attention allocation using fuzzy theory and quantified the selective attention mechanism of the information using hybrid entropy [41,42]. Wanyan et al. [15] and Wu et al. [16] applied detection efficiency factors and fatigue factors to Matsui’s fuzzy model for pilots. Considering multiple factors in the SEEV model, Wu and Wanyan developed the attention model under multi-factor conditions [17]. This was an attempt to integrate the SEEV model and the fuzzy model. Based on subjective expected utility theory (SEU), a human is an optimal information processing processor [43]. The comprehensive consideration of the theory aimed to maximize the acquisition of the important information and minimize the fuzziness of the scene. The above attention allocation models based on fuzzy theory usually involved two main factors: information value and information fuzziness [15,16,17,41,42]. 

The above models used the membership degrees of the importance of the information (value: 0–1) expressing the information value. However, the drawback of the application of membership degrees without processing was that the attention allocation ratio did not increase when the information value increased. This means that a high information value might not lead to a high attention allocation ratio. In this aspect, the above models based on fuzzy theory need to be improved. In this study, we tried to solve this problem and demonstrate that our improvement is reasonable and effective.

The proposed attention allocation model was built based on the work of Matsui’s and Wanyan et al.’s models [15,16,42]. The information value is presented by the proposed value priority function using the membership degrees of the importance and information amounts. Using the theory of hybrid entropy, the proposed model expresses the supervisors’ fuzzy cognition of the information processing in the human brain. Combining these two cognitive processes, an increasing attention allocation model was built along with the increasing information value. The BAS system is a typical interface used by supervisors to monitor the environmental equipment in subway systems. We conducted an experiment using a BAS simulator, which showed that the proposed model is effective. Compared with Matsui’s and Wanyan’s model, the proposed model has several advantages and reasonable improvements. We think that our proposed model has potential for applications in behavior analysis, cognitive optimization, and industrial ergonomic design.

## 3. Methods

### 3.1. Value Priority Function

The supervisory task involves monitoring and controlling a large amount of system information. The information on the monitors can be partitioned into several visual displays and independent meaningful sections, creating *I_i_*:

(1) $$I_{i}=\left(I_{1},I_{2},\ldots ,I_{n}\right)$$

The attention allocation model aims to predict the attention behavior of the supervisor. The attention allocation is the ratio *A_i_* of the virtual attention time required to focus on the information *I_i_* to the total virtual attention time for the whole task, as shown in Equation (2). The proposed attention allocation model aims to build the mapping relationship between *I_i_* and *A_i_* before the supervisory task:

(2) $$A_{i}=\left(A_{1},A_{2},\ldots ,A_{n}\right)$$

Based on the research of Wickens, the attention behaviors of a skillful operator are rarely affected by the bottom-up channel unless the bottom-up factors have independent meaning [11]. Subsequent research supported this view [14]. Thus, the extension of this theory tried to consider multiple factors in particular scenes. 

During a familiar procedural task, the supervisor of the system would have previously evaluated the information value based on their knowledge and training. However, the priority is fuzzy to recognize. Based on fuzzy theory, the membership degree of the information importance is considered the information value *V_i_* to every information *I_i_*, as shown in Equation (3). For a task, the membership degrees of the importance for the informative sections are certain values. Usually, the values are provided by experts in the field who are familiar with the task [15,16]:

(3) $$V_{i}=\left(V_{1},V_{2},\ldots ,V_{n}\right)$$

Matsui and Wanyan et al. considered these membership degrees as the information value [15,16,42]. The possible values are 0–1. In this research, we wanted to build a visual attention allocation model with a higher attention ratio to the higher information value. Therefore, the information value, *V_i_*, needed to be improved to value the priority of the information, *V_i_’*, which ranges from 0 to positive infinity.

Considering supervisors as the information processor, the information value *V_i_* of the sections should be converted with its information entropy. Usually, the information amount, *H_i_*, in Equation (4), presents the information sections when event *i* occurs, which is related to the probability that the certain information *Pr_i_* occurs:

(4) $$H_{i}=-\ln Pr_{i}$$

The definition of information amounts shows:

(1) The information amount is monotonically decreases, which means that a low probabilistic event occurs with a high amount of information.
(2) The information amount tends to be 0 when the probability of the occurrence of the event tends to be 1, which means that the inevitable event carries no amount of information.

The improvement in the information value *V_i_* needs to consider the following cognitive behaviors:

(1) Supervisors pay more attention to information sections with a higher information value. This means that the ratio of the attention allocation is a monotonic increasing function of the information value.
(2) The ratio of the attention allocation to the information (from 0 to 1) tends to be 1 when the information value (from 0 to 1) tends to be 1. This means that the information valued at 1, places the highest requirements on attention resources. If the supervisor transfers their attention to other information sections, it leads to a serious failure.

Referring to similarities to the definition of information amounts and cognitive behaviors, we propose a value priority function F(*V_i_*), to manage the information value *V_i_*. The improved information value is value priority *V_i_*’, as shown in Equation (5), and represents the tendency where supervisors tend to pay more attention to the more important information:

(5) $$V_{i}^{\prime }=F\left(V_{i}\right)=-\ln\left(1-V_{i}\right)$$

### 3.2. Information Fuzziness Tendency

The psychological and physiological states of the supervisor affect attention behavior. *P_i_* represents the probability that the supervisor will correctly process the information (Equation (6)). When they have a higher probability of correctly evaluating the information, the supervisor pays more attention to this information [15,16]:

(6) $$P_{i}=\left(P_{1},P_{2},\ldots ,P_{n}\right)$$

This uncertain evaluation of the information *P_i_* is caused by the fuzzy information value *V_i_*. Based on fuzzy theory, the ambiguities of information can be quantified by hybrid entropy. The hybrid entropy *S* represents the cognition fuzzy level, which involves the informative probabilistic entropy *H_prob_* and the informative binary entropy *H_bin_*:

(7) $$\begin{array}{c} S=H_{prob}+H_{bin}=\sum_{i=1}^{n}-P_{i}\ln P_{i}+\sum_{i=1}^{n}P_{i}h\left(V_{i}\right)\\ \;|h\left(V_{i}\right)=-V_{i}lnV_{i}-\left(1-V_{i}\right)ln\left(1-V_{i}\right) \end{array}$$

The supervisor is the optimal processer of the information when they have the highest attention cognition. That is, the best cognitive state occurs when the hybrid entropy *S* reaches the maximum. The supervisor can process the most amount of information they can based on SEU theory [43] and in that case, the *S* will reach the *S_max_*. On this condition, *S* = *S_max_*, we calculated the probability of the correct evaluation of *P_i_* based on Equation (7) using the Lagrange multiplier with constraints. Finally, the critical points *P_i_* was calculated using Equation (8). The calculation of the critical points can be found in the current research [15]:

(8) $$P_{i}=\frac{\exp h\left(V_{i}\right)}{\sum_{i=1}^{n}\exp h\left(V_{i}\right)},\;\mathrm{For}\;S\;\mathrm{reaches}\;\mathrm{the}\;\mathrm{maximum}|S=S_{max}$$

When the hybrid entropy *S* reaches the maximum, humans become the best processer of information based on the maximum entropy principle. This means that the human optimally processes the information to decrease the uncertainty of the HMI. *S_max_* quantifies this ability, called mental entropy (ME).

The probability of the correct evaluation *P_i_* presents the tendency of supervisors to pay more attention to more fuzzy information [15,16].

### 3.3. Attention Allocation Model

According to the above-mentioned analysis, the cognitive process of the information in the supervisory task involves two channels. The supervisors process the information value based on their previous cognition and knowledge, while they process the information fuzziness based on the psychological and physiological state of the supervisor. Combining these two channels, we can obtain the information cognitive evaluation *C_i_* using Equation (9). Finally, the cognitive process is defined by the probability of the correct evaluation *P_i_* and the information value *V_i_*:

(9) $$C_{i}=P_{i}V_{i}^{\prime }=P_{i}F\left(V_{i}\right)=-P_{i}\ln\left(1-V_{i}\right)$$

Kleinman defined the attention allocation *A_i_* as the ability to process the information [44]. Based on information science, he considered humans the optimal multiple processors to process the information channel *I_i_*. The subsequent research adopted this idea as the foundation of the attention allocation model and defined the attention allocation *A_i_*, which showed that the information cognitive evaluation *C_i_* determines the final attention allocation strategy. The final attention allocation model for the supervisors can be represented as:

(10) $$A_{i}=\frac{C_{i}}{\sum_{i=1}^{n}C_{i}}=\frac{-P_{i}\ln\left(1-V_{i}\right)}{\sum_{i=1}^{n}-P_{i}\ln\left(1-V_{i}\right)}$$

**Figure 1:**
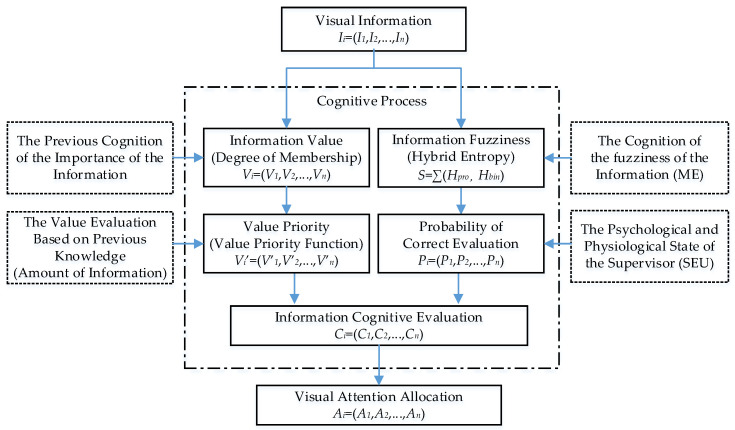
The framework of the proposed visual attention allocation model for the supervisors.

Figure 1 shows the framework of the proposed visual attention allocation model for the supervisors and shows how to build the model and the dependent theories.

## 4. Experiment

### 4.1. Apparatus

**Figure 2:**
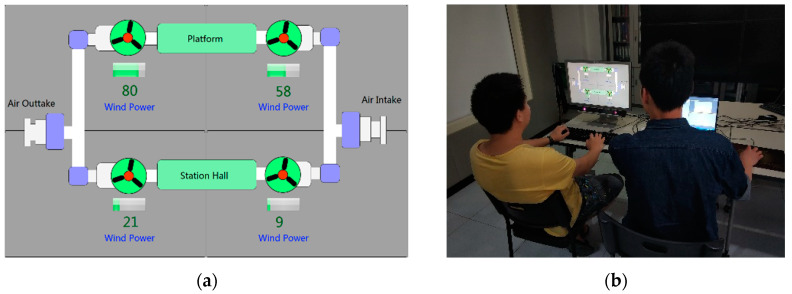
(**a**) The human-machine interface of the Building Automatic System (BAS). (**b**) The experiment environment.

The experiment interface was a simulator running the BAS system showing the statuses of the main air exchange fans in the subway system (Figure 2a). The system information was shown on a 22-inch digital screen with a resolution ratio of 1680 × 1050. Based on capturing the reflected infrared lights with the eyes, the SMI RED500 (Silicon Microstructures Inc., California, CA, USA) tracked the participant’s eye movements with a 60Hz infrared-based camera. We used it to record the participant’s visual behaviors including the gaze points on the screen, the fixation distribution. The experiment environment is shown in Figure 2b.

### 4.2. Participants

Fourteen students from the Beijing Jiaotong University, Beijing, China participated in the study (seven men, seven women, 25.3 ± 2.6 years old). All participants were familiar with the operation of a computer keyboard and had background knowledge of the subway operation. All participants were right-handed with normal vision.

### 4.3. Experimental Task

The BAS interface showed four main sections for four air fans in a fire scene. During the task, the participants needed to monitor the four speed indicators of the air fans and allocated their attention resources based on the pre-given membership degrees of the importance of the four sections. The speeds of the air fans continuously changed every second which was shown in the indicators. When the indicators showed an excess speed of the fans (>80% rated), the participants had to press the corresponding key (Insert, Delete, Home, or End for the four sections) on the keyboard to control its speed for overload protection. The abnormal excess speed would remain for one second. If the participants missed it or entered the wrong response to the overload air fans, they would be considered as not having paid attention to the corresponding section on the screen. The accuracy rates and eye behaviors were recorded during the whole task. We used the keys Insert, Delete, Home, and End, because the layout of these four keys is similar to the HMI of the BAS simulator.

The correct response to the abnormal section results in a corresponding score point based on the membership degrees of the importance, e.g., a correct response to areas of interest (AOI) 0.9 will get 0.9 points. It is obvious that response to the section which has higher information value and responses to more abnormal sections will get a higher total score point. The goal of the participants is to achieve the highest total score points.

### 4.4. Experimental Procedure

The operation of the BAS interface was explained to the participants. At first, the membership degrees of the importance of the four air fans were set based on their relative priorities in a fire scene. The participants were instructed to remember and understand the membership degrees given the possibility that the system would encounter a serious failure if the supervisor missed the overload control. Participants were asked to practice task operations twice to simulate the supervisor’s experience and previously acquired knowledge. Through practice, the participants became familiar with the operation of the BAS and the functioning of the system. They would not need to look at the keyboard when they pressed the keys.

During the formal experiment, the participants were asked to complete the calibration process for the eye tracking devices first. Then, they were asked to freely allocate their attention to the four sections. They need to try their best to response to all the abnormal sections in the HMI. The test continued for five minutes and during the whole test eye behaviors were recorded.

### 4.5. Data Analysis

The experimental results of the key-press response showed that the sections had a different correct response ratio, *O_i_*, which was calculated by the number of correct responses and total overload occurrences during this section. The correct response to the overload section was considered as selective attention to the corresponding section. Therefore, the fractional attention, *A_k_i_* (key), was quantified by the experimental key-press data as:

(11) $$A_{k\_i}=\frac{O_{i}}{\sum_{i=1}^{n}O_{i}},\left(i=1,2,3,4\right)$$

After the experiment, the participants’ eye tracking data were analyzed using the eye behavior analysis software Begaze, which was developed by Silicon Microstructures Inc., California, CA, USA. In Begaze, the four sections were identified by the four areas of interest (AOIs). The fixation behaviors of the different AOIs were extracted from the original data, which meant that the participants paid attention to the corresponding sections. Based on the fixation times, *m_i_,* for a certain AOI, the fractional attention, *A_e_i_* (eye), was quantified by the experimental eye tracking data with:

(12) $$A_{e\_i}=\frac{m_{i}}{\sum_{i=1}^{n}m_{i}},\left(i=1,2,3,4\right)$$

Using Equation (10), the theoretical results of the proposed supervisors’ visual attention allocation model could be calculated as:

(13) $$A_{p\_i}=\frac{-P_{i}\ln\left(1-V_{i}\right)}{\sum_{i=1}^{n}-P_{i}\ln\left(1-V_{i}\right)},\left(i=1,2,3,4\right)$$

Matsui’s and Wanyan’s model was used as a comparison model; their model was used for aircraft pilots [15,42]. The theoretical results of their model can be calculated using Equation (14). This model is referred to as the Matsui’s Model, as he was the first to create the basic method:

(14) $$A_{m\_i}=\frac{P_{i}V_{i}}{\sum_{i=1}^{n}P_{i}\ln V_{i}},\left(i=1,2,3,4\right)$$

The experiment aimed to compare *A_k_i_* (Key), *A_e_i_* (Eye), and *A_p_i_* (Proposed) and *A_m_i_* (Matsui’s). We adopted the SPSS 25.0 statistics software (developed by IBM, California, CA, USA) to process the data. The results are expressed as the mean ± standard deviation (m ± s). Bivariate Pearson correlation analysis was used to analyze the relationship between the theoretical results and the experimental models. Considering the main difference between the Matsui’s Model and the proposed model, the one-sample T test was used to analyze the difference between the two experimental results and the two theoretical results at the sections that had a high membership degree of importance.

## 5. Results

### 5.1. Theoretical and Experimental Results 

Through the information value, *V_i_*, pre-given by the experts for the four sections, in one scene the section of the air intake fan in the station hall (intake@hall) had 0.1 membership degrees of information importance, the section of the air outtake fan in the station hall (outtake@hall) had 0.3; and the section of the air outtake fan in the platform (outtake@platform) had 0.7. The section of the air intake fan in the platform (intake@platform) had 0.9 membership degrees of information importance. 

The fractional attention, *A_i_* (%), of each section can be predicted by both Matsui’s Model, *A_m_i_*, and the proposed model, *A_p_i_*. The theoretical values are shown in Table 1. There was a significant difference between the two models in the section that had a high membership degree of importance. The proposed model, *A_p_i_*, monotonically increased with the information value, *V_i_*, while Matsui’s Model, *A_m_i_*, did not. 

The experimental results of the key-press response are shown in Table 2. The key press results showed that a higher information value, *V_i_*, led to a higher correct response ratio, *O_i_*. This indicted that supervisors paid more attention to the information that had a higher information value, *V_i_*, and obtained a higher ratio of correct responses, *O_i_*.

The experimental results of the eye tracking are shown in Table 3. The results showed a similar attention tendency as the key-press results. A higher information value, *V_i_*, led to more fixation points on the higher-value sections.

**Figure 3:**
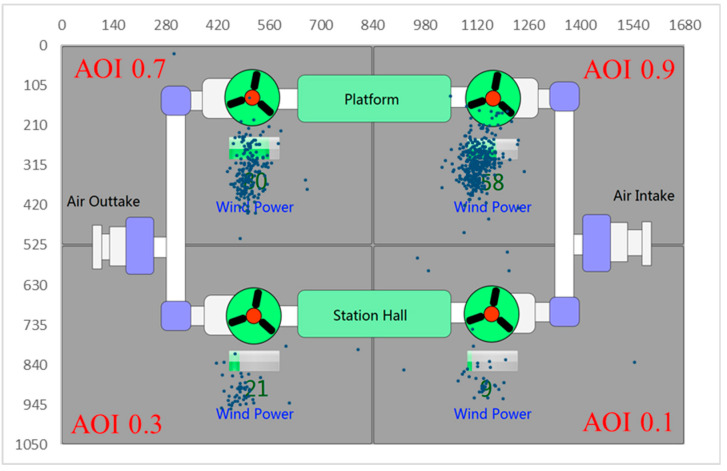
Fixation points of the eye tracking data on the screen.

The eye tracking results provided the most practical evidence of the supervisors’ attention allocation strategy. Figure 3 shows the fixation points of one participant. The figure shows that the participant paid more attention to the section that had a higher information value, *Vi* (AOI 0.9 > AOI 0.7 > AOI 0.3 > AOI 0.1).

### 5.2. Comparison of Theoretical and Experimental Results

**Figure 4:**
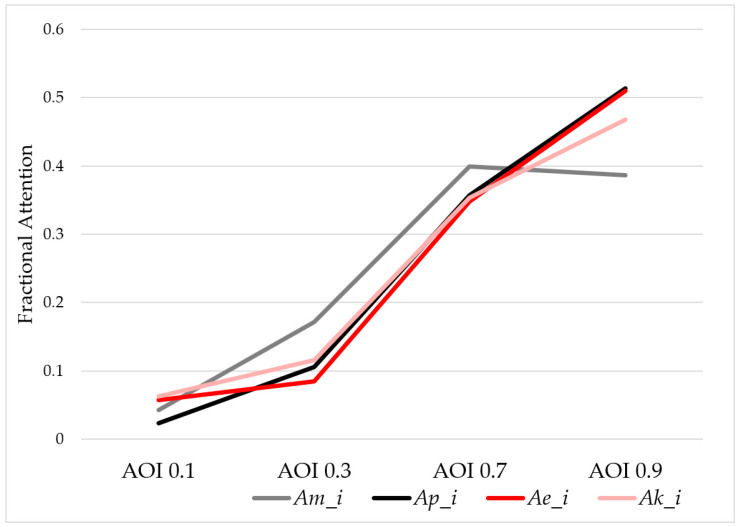
Comparison of the theoretical and experimental results.

The fractional attention values of the key-press response experiment and the eye movement tracking experiment as well as the two theoretical values are shown in Figure 4.

As Figure 4 shows, the experimental results better supported the proposed model compared to Matsui’s Model. The correlation analysis between the four results were processed and the results are shown in Table 4, which shows that the proposed model was significantly associated with the participants’ experimental behaviors in both Key Press and Eye Tracking (*P* < 0.01). The two experimental behaviors, Key Press and Eye Tracking, were significantly correlated (*P* < 0.01), the two experimental results showed coincident behaviors, confirming that the data analysis method is effective. We also found that the correlation between Matsui’s Model and the proposed model was 0.939, which means that these two models were close but different. The proposed model was more effective.

Based on the method used in the proposed model, the significant difference between the two theoretical models were observed for AOI 0.7 and 0.9. The T-test was used to analyze the difference. The results of the statistics are shown in Table 5. 

The statistics showed that the experimental Eye Tracking and Key Press results were not significantly different (*P* > 0.05) from the proposed model at AOI 0.7, but were different from Matsui’s Model at AOI 0.7. 

For AOI 0.9, the experimental Key Press result showed a significant difference with the proposed model because the participants may not respond to the AOI 0.9 section, even if the participants focused on the section while the overload scene for AOI 0.9 was random. However, the eye tracking results showed no significant difference *(P* > 0.05) with the proposed model, which is more practical. 

For Matsui’s Model, the experimental results showed a significant difference for AOI 0.7 and AOI 0.9.

## 6. Discussion

### 6.1. Discussion of the Value Priority Function

The experimental results showed that the proposed model predicts supervisors’ visual attention allocation more accurately than Matsui’s Model. The improvement in the results from the proposed model was in the high information value, *V_i_*, which was due to the proposed value priority function, F(*V_i_*), in Equation (5). The role of this function is discussed in depth below.

The proposed value priority function, F(*V_i_*), processes the information value, *V_i_,* and the processed value is *V_i_’*. The proposed model used *V_i_’* to present the value priority, whereas Matsui’s Model uses the original information value, *V_i_*. This finally affected the information cognitive evaluation, *C_i_*, process. Therefore, the two theoretical models are based on a different information cognitive evaluation, *C_i_*. The fractional cognitive evaluation in Matsui’s Model, *C_m_i_*, and the proposed model, *C_p_i_*, can be calculated using Equations (15) and (16), respectively:

(15) $$C_{m\_i}=P_{i}V_{i}$$

(16) $$C_{p\_i}=P_{i}V_{i}^{\prime }=P_{i}F\left(V_{i}\right)=-P_{i}\ln\left(1-V_{i}\right)$$

Assume that the number of the independent information sections, *i*, reaches infinity. Assuming that the corresponding information value, *V_i_* (membership degree of the importance), ranges from 0 to 1, the probability of the correct evaluation, *P_i_*, can be calculated using Equation (17) based on Equation (8):

(17) $$P_{i}=\frac{\exp h\left(V_{i}\right)}{\int_{V_{i}=0,i=0}^{V_{i}=1,i=\infty }\exp h\left(V_{i}\right)}$$

**Figure 5:**
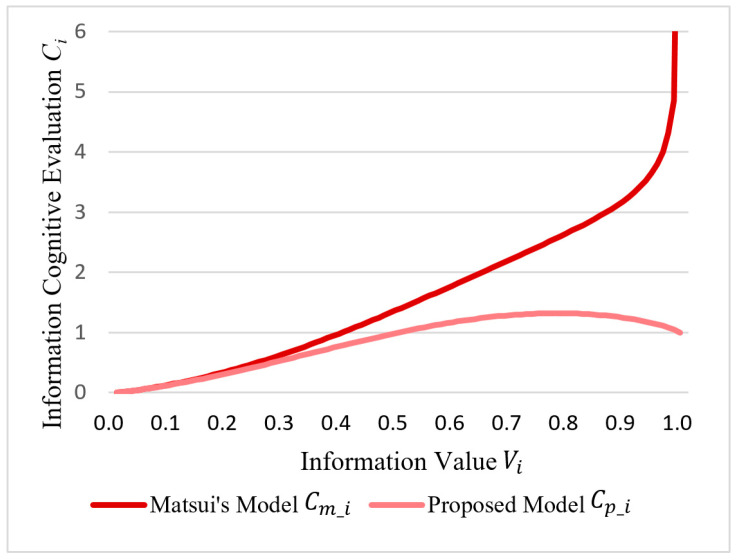
The information cognitive evaluation *C_i_* based on Matsui’s Model, *C_m_i_*, and the proposed model, *C_p_i_*, along with information value, *V_i_*.

Along with the information value, *V_i_*, the information cognitive evaluation, *C_i_*, values based on Matsui’s Model, *C_m_i_*, and the proposed model, *C_p_i_*, are shown in Figure 5.

As the figure shows, the proposed model is more reasonable than Matsui’s Model in the following aspects:

(1) The proposed *C_p_i_* monotonically increases along with the information value, *V_i_,* which is more reasonable in practice. The supervisor pays more attention to the section that has a higher information value; the supervisor is afraid of missing the most important information that may have a great probability to cause a system failure.
(2) When the information value, *V_i_*, tends to be 1, the information cognitive evaluation of the proposed *C_p_i_* tends to be infinity, but Matsui’s *C_m_i_* showed a convergent value (Equation (18)). In practice, the highest information value (*V_i_* = 1) (the membership degree of the information importance is 100%) means that the model is absolutely important and the supervisor cannot miss it. For this point, the proposed model has rationality:
  (18) $$C_{m{\_}_{\infty }}=\lim_{V_{i}\to 1,i\to \infty }C_{m_{i}}=0.006\;C_{p\_\infty }=\lim_{V_{i}\to 1,i\to \infty }C_{p\_i}=\infty$$
(3) The proposed *C_p_i_* increases after 0.7822 along with the information value, *V_i_*. However, Matsui’s *C_m_i_* decreases after 0.7822, which means that a high information value above 0.7822 will not lead to a higher information cognitive evaluation status (Equation (19)), which is not realistic. Therefore, our proposed value priority function, F(*V_i_*), is an improvement that corrects the unreasonable part of Matsui’s Model:
  (19) $${C_{m\_i}}^{\prime }=\exp((V_{i}-1)\ln(1-V_{i})-V_{i}\ln(V_{i}))(\ln(1-V_{i})-\ln(V_{i}))\;{C_{m\_i}}^{\prime }=0|V_{i}=0.7822$$
(4) As the overall curve of the proposed *C_p_i_* becomes steeper, the attention allocation of the supervisor tends to be more concentrated, and the adjustment of the supervisors’ attention allocation is more reasonable.

### 6.2. Discussion of Attention Allocation Models

The proposed value priority function, F(*V_i_*), affects the information cognitive evaluation, *C_i_*; *C_i_* affects the whole visual attention allocation model, *A_i_*. The difference between Matsui’s and the proposed model in theory is discussed in depth below.

Based on the different information cognitive evaluation models, *C_i_*, the fractional attention in Matsui’s Model, *A_m_i_*, and the proposed allocation model, *A_p_i_*, can be calculated using Equations (20) and (21) based on Equation (10), respectively:

(20) $$A_{m\_i}=\frac{C_{m\_i}}{\int_{V_{i}=0,i=0}^{V_{i}=1,i=\infty }C_{m\_i}}=\frac{P_{i}V_{i}}{\int_{V_{i}=0,i=0}^{V_{i}=1,i=\infty }P_{i}V_{i}}$$

(21) $$A_{p\_i}=\frac{C_{p\_i}}{\int_{V_{i}=0,i=0}^{V_{i}=1,i=\infty }C_{p\_i}}=\frac{P_{i}V_{i}^{\prime }}{\int_{V_{i}=0,i=0}^{V_{i}=1,i=\infty }P_{i}V_{i}^{\prime }}=\frac{P_{i}F\left(V_{i}\right)}{\int_{V_{i}=0,i=0}^{V_{i}=1,i=\infty }P_{i}F\left(V_{i}\right)}=\frac{-P_{i}\ln\left(1-V_{i}\right)}{\int_{V_{i}=0,i=0}^{V_{i}=1,i=\infty }-P_{i}\ln\left(1-V_{i}\right)}$$

**Figure 6:**
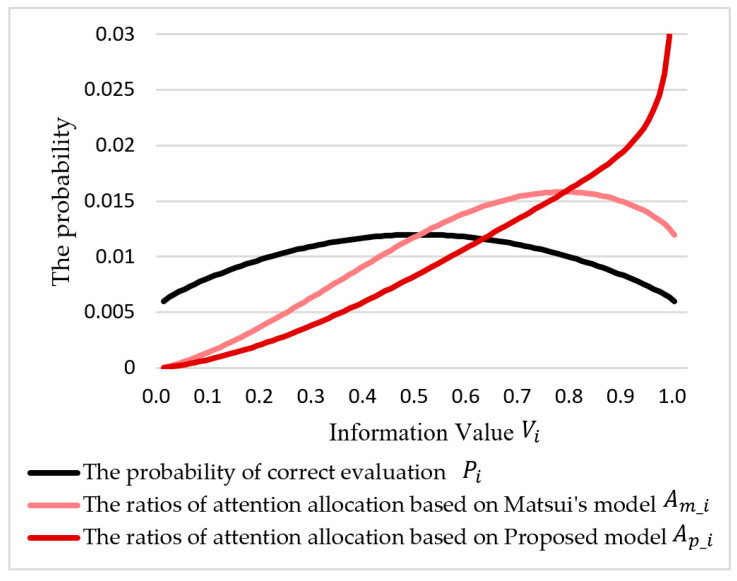
The ratios of attention allocation based on Matsui’s Model, *A_m_i_*, and the proposed model, *A_p_i_*, and the probability of the correct evaluation, *P_i_*, along with the information value, *V_i_.*

Along with the information value, *V_i_*, the attention allocation based on Matsui’s Model, *A_m_i_*, and the proposed model, *A_p_i_*, is shown in Figure 6. Based on Equation (17), we added the probability of the correct evaluation *P_i_* into the figure. *P_i_* is a factor of information fuzziness tendency, which affects the model.

As the figure shows, the proposed model, *A_p_i_*, and Matsui’s Model, *A_m_i_*, are significantly different:

(1) The proposed model, *A_p_i_*, monotonically increased along with the information value, *V_i_;* but the Matsui’s model did not. The supervisor pays more attention to the section that has a higher information value *V_i_*;
(2) The Matsui’s attention allocation model has a critical value at 0.7822, calculated using Equation (22). This means that a higher information value, *V_i_*, may not lead to a higher attention allocation ratio, *A_i_*. The highest information value ($V_{i}\to 1$) will not acquire the supervisors’ entire visual attention ($A_{i}\to 100\%$):
  (22) $${A_{m\_i}}^{\prime }=\exp((V_{i}-1)\ln(1-V_{i})-V_{i}\ln V_{i})+V_{i}\exp((V_{i}-1)\ln(1-V_{i})-V_{i}\ln V_{i})(\ln(1-V_{i})-\ln V_{i})\;{A_{m\_i}}^{\prime }=0|V_{i}=0.7822$$
(3) The probability of the correct evaluation, *P_i_*, reaches the highest value when the information value, *V_i_*, = 0.5 (Equation (23)), which means that the supervisor has a higher successful probability to process the information in the visual section that has medium information value, *V_i_*:
  (23) $$P_{0}=\lim_{V_{i}\to 0,i\to 0}P_{i}=0.006\;P_{m}=\lim_{V_{i}\to 0.5,i\to mid}P_{i}=0.012\;P_{\infty }=\lim_{V_{i}\to 1,i\to \infty }P_{i}=0.006$$
(4) The proposed attention allocation model is not significantly different from Matsui’s Model before the intersection near the critical point in Matsui’s Model. After the intersection, the ratio of the attention allocation tended to be a steep curve. This means that the participants focused on the highest value information.

In summary, the proposed model is more reasonable and effective, as shown through the above analysis. The experimental results supported the above theoretical discussion. The proposed model is an accurate quantitative method that can be used to analyze the attention allocation strategy of supervisors.

The proposed model can basically quantify attention allocation using hybrid entropy. The other current models based on Matsui’s Model, which consider the fatigue, effort, salience, and information detection efficiency [15,16,17], can replace the basic Matsui Model with the proposed model to improve results. The above factors were weakened in the experiment in this article on purpose to highlight the research achievement that prevented it from being overwhelmed by the above factors.

## 7. Conclusions

By referencing the definition of the information amounts, the value priority function was proposed in this paper. Considering supervisors as information processors, the information fuzziness was quantified based on hybrid entropy theory. Supervisors tend to pay more attention to important and fuzzy information. Combining these two aspects, a quantitative visual attention allocation model for supervisors was built. The experiment showed that the proposed model was more effective than the current model. The difference between the proposed theory and the current theory was further discussed, which showed that the proposed model has mathematical specialties that coincide more with practical applications and compensated for the deficiency in the current model. 

Further Application: Using the proposed model, visual attention behavior can be predicted before the task. This will help researchers analyze supervisors’ behaviors and evaluate the ergonomics of the HMI. The risk of cognitive deficits can be detected early, and targeted attention training can help supervisors schedule limited behavioral resources. Optimizing the HMI design with human behavior will make the system safer and more efficient.

## Figures and Tables

**Table 1:** Information value *V_i_* and theoretical values of Matsui’s model *A_m_i_*, proposed model *A_p_i_*.

Sections on the HMI	Intake@hall	Outtake@hall	Outtake@platform	Intake@platform
*V_i_*	0.1	0.3	0.7	0.9
*Am_i*	4.29	17.13	39.97	38.61
*Ap_i*	2.35	10.58	35.73	51.34

**Table 2:** Experimental values based on the key press response data.

Sections on the HMI	Intake@hall	Outtake@hall	Outtake@platform	Intake@platform
*V_i_*	0.1	0.3	0.7	0.9
*O_i_*	0.12 ± 0.08	0.23 ± 0.09	0.67 ± 0.08	0.89 ± 0.06
*A_k_i_*	6.29 ± 3.92	11.56 ± 4.22	35.34 ± 5.23	46.81 ± 3.79

**Table 3:** Experimental values based on the eye tracking data.

Sections on the HMI	Intake@hall	Outtake@hall	Outtake@platform	Intake@platform
*V_i_*	0.1	0.3	0.7	0.9
*A_e_i_*	5.77 ± 4.50	8.47 ± 3.91	34.78 ± 6.20	50.98 ± 7.80

**Table 4:** The correlation between models.

		Matsui’s Model	Proposed Model	Key Press	Eye Tracking
**Matsui’s Model**	Pearson Correlation	1	0.939	0.912	0.944
	Sig. (2-tailed)		0.061	0.088	0.056
**Proposed Model**	Pearson Correlation	0.939	1	0.995 *	0.998 *
	Sig. (2-tailed)	0.061		0.005	0.002
**Key Press**	Pearson Correlation	0.912	0.995 *	1	0.996 *
	Sig. (2-tailed)	0.088	0.005		0.004
**Eye Tracking**	Pearson Correlation	0.944	0.998 *	0.996 *	1
	Sig. (2-tailed)	0.056	0.002	0.004	

* Correlation was significant at the 0.01 level (two-tailed).

**Table 5:** The one-sample T-test between the models at areas of interest (AOI) 0.7 and 0.9.

Models	Experimental Results	t	Sig. (2-tailed)	Mean Difference	95% Confidence Interval of the Difference	
					Lower	Upper
Proposed Model @0.7 Test Value = 0.3573	EyeTracking@0.7	−0.551	0.592 *	−0.00948	−0.0470	0.0280
	KeyPress@0.7	−0.270	0.792 *	−0.00392	−0.0356	0.0277
Proposed Model @0.9 Test Value = 0.5134	EyeTracking@0.9	−0.168	0.869 *	−0.00365	−0.0508	0.0435
	KeyPress@0.9	−4.317	0.001	−0.04534	−0.0682	−0.0225
Matsui’s Model @0.7 Test Value = 0.3997	EyeTracking@0.7	−3.015	0.011	−0.05188	−0.0894	−0.0144
	KeyPress@0.7	−3.189	0.008	−0.04632	−0.0780	−0.0147
Matsui’s Model @0.9 Test Value = 0.3861	EyeTracking@0.9	5.712	0.000	0.12365	0.0765	0.1708
	KeyPress@0.9	7.803	0.000	0.08196	0.0591	0.1048

* Significance level is at the 0.05 level (two-tailed).
